# Age-dependent phenotypic and molecular evolution of pediatric MDS arising from GATA2 deficiency

**DOI:** 10.1038/s41408-025-01309-6

**Published:** 2025-07-15

**Authors:** Lili Kotmayer, Emilia J. Kozyra, Guolian Kang, Brigitte Strahm, Ayami Yoshimi, Sushree S. Sahoo, Victor B. Pastor, Enrico Attardi, Rebecca Voss, Luca Vinci, Max Kaiser, Michael N. Dworzak, Barbara De Moerloose, Martina Sukova, Jan Starý, Henrik Hasle, Kirsi Jahnukainen, Sophia Polychronopoulou, Krisztián Kállay, Owen P. Smith, Andrea Malone, Shlomit Barzilai Birenboim, Riccardo Masetti, Jochen Buechner, Marek Ussowicz, Paula Kjöllerström, Ivana Bodova, Marko Kavcic, Albert Català, Dominik Turkiewicz, Markus Schmugge, Valerie de Haas, Victoria I. Okhomina, Cristian Sotomayor, Paula Catalán, Claudia Wehr, Ulrich Salzer, Ulrich Germing, Norbert Gattermann, Csaba Bödör, Nathan Gray, Sara Lewis, Akiko Shimamura, Alessandra Giorgetti, Miriam Erlacher, Charlotte M. Niemeyer, Marcin W. Wlodarski

**Affiliations:** 1 Department of Hematology, St. Jude Children’s Research Hospital, Memphis, USA; 2 Division of Pediatric Hematology and Oncology, Department of Pediatrics and Adolescent Medicine, Medical Center, Faculty of Medicine, University of Freiburg, Freiburg, Germany; 3 Department of Biostatistics, St. Jude Children’s Research Hospital, Memphis, USA; 4 Department of Pathology, St. Jude Children’s Research Hospital, Memphis, USA; 5 Department of Biomedicine and Prevention, Molecular Medicine and Applied Biotechnology, University of Rome Tor Vergata, Rome, Italy; 6 St. Anna Children’s Hospital, Department of Pediatrics and Adolescent Medicine, Medical University of Vienna, Vienna, Austria; 7 St. Anna Children’s Cancer Research Institute, Vienna, Austria; 8 Department of Pediatric Hematology and Oncology, Ghent University Hospital, Ghent, Belgium; 9 Department of Pediatric Hematology and Oncology, 2nd Faculty of Medicine, Charles University and University Hospital Motol, Prague, Czech Republic; 10 Department of Pediatrics and Adolescent Medicine, Aarhus University Hospital, Aarhus, Denmark; 11 Children’s Hospital, University of Helsinki and Helsinki University Hospital, Helsinki, Finland; 12 Department of Paediatric Hematology-Oncology, Aghia Sophia Children’s Hospital, Athens, Greece; 13 Pediatric Hematology and Stem Cell Transplantation Department, Central Hospital of Southern Pest, Budapest, Hungary; 14 Children’s Health Ireland, Dublin, Ireland; 15 Trinity College Dublin, Dublin, Ireland; 16 Pediatric Hematology-Oncology, Schneider Children’s Medical Center and Faculty of Medical & Health Sciences, Tel Aviv University, Petah Tikva, Israel; 17 IRCCS Azienda Ospedaliero Universitaria di Bologna, Bologna, Italy; 18 Oslo University Hospital, Department of Pediatric Hematology and Oncology, Oslo, Norway; 19 Department of Paediatric Bone Marrow Transplantation, Oncology and Hematology, Wroclaw Medical University, Wroclaw, Poland; 20 Unidade de Hematologia Pediátrica, Hospital Dona Estefânia, ULS São José, Lisboa, Portugal; 21 Bone Marrow Transplantation Unit, Department of Pediatric Hematology and Oncology, National Institute of Children’s Diseases, Bratislava, Slovakia; 22 University Medical Center Ljubljana, Ljubljana, Slovenia; 23 Department of Hematology and Oncology, Hospital Sant Joan de Déu, Barcelona, Spain; 24 Department of Pediatric Oncology and Hematology, University Hospital in Scania, Lund, Sweden; 25 University Children’s Hospital Zurich, Zürich, Switzerland; 26 Prinses Maxima Centre, Utrecht, The Netherlands; 27 Section of Hematology, Oncology and Stem Cell Transplantation, Division of Pediatrics, Pontificia Universidad Católica de Chile, Santiago, Chile; 28 Bone Marrow Transplantation Unit, Hospital Dr. Luis Calvo Mackenna, Santiago, Chile; 29 Department of Medicine I: Hematology, Oncology and Stem Cell Transplantation, Medical Center, Faculty of Medicine, University of Freiburg, Freiburg, Germany; 30 Department of Rheumatology and Clinical Immunology, Medical Center, Faculty of Medicine, University of Freiburg, Freiburg, Germany; 31 Center for Chronic Immunodeficiency, Medical Center, Faculty of Medicine, University of Freiburg, Freiburg, Germany; 32 Department of Hematology, Oncology and Clinical Immunology, Heinrich-Heine-Universität, Düsseldorf, Germany; 33 HCEMM-SE Molecular Oncohematology Research Group, MTA-SE Lendulet Molecular Oncohematology Research Group, Department of Pathology and Experimental Cancer Research, Semmelweis University, Budapest, Hungary; 34 Dana-Farber and Boston Children’s Cancer and Blood Disorders Center, Harvard Medical School, Boston, USA; 35 Regenerative Medicine Program, Bellvitge Institute for Biomedical Research (IDIBELL) and Program for Clinical Translation of Regenerative Medicine in Catalonia (P-CMRC), Barcelona, Spain; 36 Department of Pathology and Experimental Therapeutics, Faculty of Medicine and Health Sciences, Barcelona University, Barcelona, Spain; 37 Department of Pediatrics and Adolescent Medicine, University Medical Center Ulm, Ulm, Germany

**Keywords:** Myelodysplastic syndrome, Cancer genetics

## Abstract

GATA2 deficiency is an autosomal dominant transcriptopathy disorder with high risk for myelodysplastic syndrome (MDS). To elucidate genotype-phenotype associations and identify new genetic risk factors for MDS, we analyzed 218 individuals with germline heterozygous *GATA2* variants. We observed striking age-dependent incidence patterns in GATA2-related MDS (GATA2-MDS), with MDS being absent in infants, rare before age 6 years, and steeply increasing in older children. Among 108 distinct *GATA2* variants (67 novel), null mutations conferred a 1.7-fold increased risk for MDS, had earlier MDS onset compared to other variants (12.2 vs. 14.6 years, *p* = 0.009) and were associated with lymphedema and deafness. In contrast, intron 4 variants exhibited reduced penetrance and lower risk for MDS development. Analysis of the somatic landscape revealed unique patterns of clonal hematopoiesis. *SETBP1* mutations occurred exclusively in patients with monosomy 7 and their frequency decreased with age. Conversely, the frequency of *STAG2* mutations and trisomy 8 increased with age and appeared protective against early development of advanced MDS. Overall, the majority (73.9%) of mutation-positive cases harbored monosomy 7, suggesting it serves as a major driver in malignant progression. Our findings provide evidence for age-appropriate surveillance, and a foundation for genotype-driven risk stratification in GATA2 deficiency.

## Introduction

GATA2 deficiency (Monarch disease ontology: 0042982, Online Mendelian Inheritance in Man [OMIM]: 601626, 614286, 614038, 614172), is an autosomal dominant transcriptopathy disorder associated with a broad phenotypic spectrum of variable severity, described over a decade ago [1–4]. This multisystem disorder carries a high risk for early-onset myeloid malignancies, specifically myelodysplastic syndrome (MDS) and acute myeloid leukemia (AML), with the median age at diagnosis estimated at 16.0-19.7 years [5–8]. Although germline *GATA2* variants are rare events in primary AML [9], GATA2 deficiency is a common driver of pediatric MDS, accounting for approximately 7% of all pediatric MDS and 15% of MDS with excess blasts (MDS-EB) [10, 11]. Non-malignant presentations arising from defect of hematopoietic stem cells include single- or multi-lineage cytopenia and immune system abnormalities ranging from mild B/NK-lymphopenia, monocytopenia to severe immunodeficiency with life-threatening infections. Accompanying constitutional features, such as lymphedema, hydrocele and sensorineural deafness, serve as frequent telltale signs of underlying germline *GATA2* variants [1–4, 7, 12]. GATA2 deficiency has a marked variation in expressivity and severity of phenotype, with a very high lifetime penetrance for immunodeficiency and MDS (reported from 37% to 100% [6, 10]). To date, several asymptomatic (“hematologically normal”) carriers have been encountered by family testing indicating either variable penetrance or delayed onset of symptoms [10, 13]. The diverse manifestations in GATA2 deficiency result from defects caused by numerous germline *GATA2* variants, which in the majority of cases arise de novo [10, 11]. *GATA2* variants fall primarily into 3 categories known to affect i) the protein structure (null variants, i.e., frameshift truncating, nonsense, silent (synonymous RNA deleterious variants), splice region, whole exon/gene deletions), ii) functionality of zinc finger 2 (ZF2) domain (missense mutations within the ZF2 domain), or iii) the allelic expression (variants in the +9.5 kb [14, 15] (intron 4) and −110 kb [16, 17] regulatory regions) [18, 19]. Mechanisms of the first 2 categories lead to *GATA2* haploinsufficiency with complete or near-complete abrogation of protein function. In contrast, variants in the regulatory regions may have a hypomorphic effect [13, 16].

Potentially due to referral bias, previous studies on GATA2 genotype-phenotype correlation have demonstrated at least in part inconsistent associations. These studies reported that: i) variants resulting in haploinsufficiency associate with lymphedema [3, 6, 20–22]; ii) null variants correlate with an earlier onset of symptoms [8, 23]; and iii) missense mutations might be predominantly associated with MDS/AML [1, 7, 10, 24]. Despite these proposed genotype-phenotype associations, it is not possible to predict the onset of MDS. MDS evolution is known to be associated with recurrent cytogenetic lesions and somatic mutations in various genes [25–27]. In GATA2-related MDS (GATA2-MDS), few studies reported a high prevalence of monosomy 7, der(1;7) [25, 26] and somatic mutations in genes including *SETBP1*, *ASXL1*, *RUNX1* [8, 24, 28–30], while somatic *STAG2* variants were rarely observed in patients with myeloid malignancy [8]. Monosomy 7 is the most common cytogenetic aberration in children with GATA2 deficiency [18].

Here, we analyzed genetic and clinical data from 218 individuals with *GATA2* variants to elucidate genotype-phenotype associations and progression patterns of GATA2-MDS. We provide new insights into the natural history of GATA2-MDS, establishing a basis for evidence-based strategies of individualized care for patients at risk.

## Materials and methods

### Patient cohort and diagnostic definitions

**Fig. 1:**
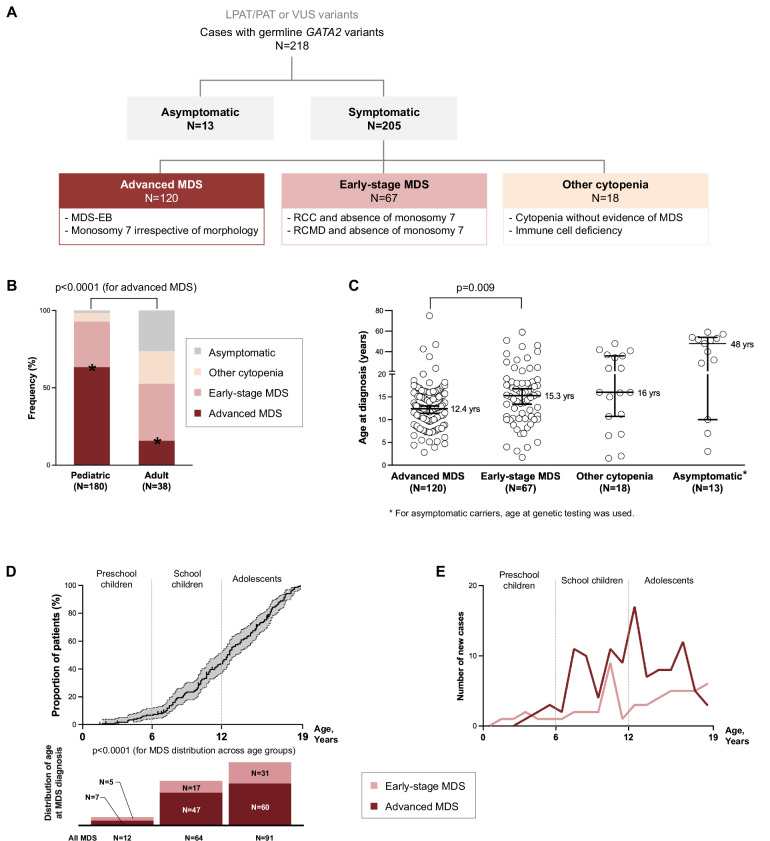
Distribution of germline GATA2-related hematological phenotypes in the study cohort. **A** Hematological phenotypes in 218 individuals (205 symptomatic and 13 asymptomatic) with confirmed germline *GATA2* variants. In symptomatic cohort, 3 clinically relevant phenotypic categories were established. Early-stage MDS encompasses cases with refractory cytopenia of childhood and refractory cytopenia with multilineage dysplasia in adults. Details are shown in Table S1. **B** Frequency of phenotypes compared between pediatric and adult patients (*N* = 218, Fisher’s exact test). **C** Age at diagnosis across different phenotypic categories (*N* = 218, Non-parametric Mann-Whitney U test). **D** Time to disease in pediatric GATA2-MDS cohort (*N* = 167). By age 6 years, 7.2% of the cohort developed MDS, increasing to 45.5% by age 12 years (gray area: 95% confidence intervals). **E** Number of new diagnoses across age in pediatric GATA2-MDS cohort (advanced MDS and early-stage MDS) (*N* = 167, Chi-square test). LPAT likely pathogenic; MDS myelodysplastic syndrome, PAT pathogenic, RCC refractory cytopenia of childhood, RCMD refractory cytopenia with multilineage dysplasia, VUS variant of unknown significance.

We enrolled 218 cases with confirmed germline *GATA2* variants (Fig. 1A), using 3 pre-defined primary referral pathways: i) Diagnosis of MDS: 187 patients (167 children, 20 adults (age ≥19 years old)) consecutively enrolled in studies of European Working Group in Childhood MDS (EWOG-MDS), St. Jude Children’s Research Hospital and Dana-Farber and Boston Children’s Cancer and Blood Disorders Center, ii) Cytopenia/immunodeficiency suspicious of GATA2 deficiency: 18 patients (10 pediatric, 8 adults) referred to our diagnostic laboratories, and iii) patients undergoing family screening: 13 asymptomatic individuals (3 pediatric, 10 adults). Asymptomatic individuals were defined as *GATA2* variant carriers who did not exhibit GATA2-specific symptoms (hematologic or systemic). In these cases, screening was prompted by the diagnosis of GATA2 deficiency in an affected relative. Of the 218 cases, 132 are new, while 86 had been previously reported by us (Table S1) [10, 22, 23, 31–33].

After consensus discussions with pediatric hematologists, oncologists, transplanters, and genetic counselors with GATA2 expertise, we established 3 clinically relevant diagnostic categories for this study:

i) Advanced MDS, encompassing patients with MDS-EB [34] and any MDS subtype with acquired chromosome 7 cytogenetic abnormalities (monosomy 7 or der(1;7)), henceforth referred to as “−7”); ii) Early-stage MDS, including refractory cytopenia of childhood (RCC) and refractory cytopenia with multilineage dysplasia (RCMD) in adults, without −7 cytogenetics; iii) Other cytopenia, comprising cases with low blood counts and/or immune cytopenia/immunodeficiency with clinical characteristics and bone marrow (BM) histology insufficient for MDS diagnosis [35]. While adhering to the ICC classification of MDS [34], the categorization in our study is clinically relevant (e.g., all advanced MDS require HSCT) and allows for a more nuanced analysis of disease severity in GATA2 deficiency. Clinical and laboratory parameters were recorded and annotated according to affected organ systems. Age at diagnosis was defined by the age at MDS or cytopenia/immunodeficiency diagnosis.

### Ethics approval and consent to participate

Written informed consent was obtained from all patients or their guardians, and from family members, all in accordance with the Declaration of Helsinki. The study was approved by the institutional review board at each of the institutions (St. Jude: #NCT02720679, # Pro00006262, EWOG-MDS: #NCT00047268, #NCT00662090, #CPMP/ICH/135/95 and 430/16, MDS registry Duesseldorf: Bioregister #3973, Boston Children’s: P00021042). All applied methods and analyses were performed in accordance with relevant guidelines and regulations.

### Cytogenetic and genetic testing

Screening of the *GATA2* coding region and intron 4 (NM_032638.4) containing conserved EBOX-GATA-ETS regulatory region ( + 9.5 kb), was performed on DNA from peripheral blood at diagnosis using targeted sequencing as previously described by us [11, 23]. Germline origin of *GATA2* variants was confirmed by standard Sanger-sequencing on non-myeloid specimens (hair follicles, skin fibroblasts or purified CD3+ cells as previously reported [10, 11]) or by family segregation analysis. Allele frequency of identified germline *GATA2* variants in the control population was extracted from gnomAD v4.1.0 database (>800,000 individuals) [36]. Chromosome banding analyses for metaphase karyotyping and fluorescence-in-situ-hybridization were performed on diagnostic BM samples according to standard procedures [37]. Somatic mutations in leukemia-related genes were assessed by standard-of-care clinical testing in 65.6% (143/218) of the cases using targeted myeloid NGS panels as reported [11] (details in the supplement).

### Variant categorization and pathogenicity assessment

For analytical purposes, we classified variants into two broad functional categories: null variants and other variants. Null variants (frameshift truncating, nonsense, splice region, silent variants, whole gene deletion) represent those predicted to eliminate or severely disrupt protein function/production. Other variants (missense mutations, in-frame insertions/deletions, non-coding alterations within intron 4 regulatory region) are predicted to result in decreased expression or altered but potentially partially functional protein.

For variant pathogenicity assessment, guidelines of American College of Medical Genetics and Genomics and Association for Molecular Pathology (ACMG-AMP) were applied [38, 39]. Evidence codes allowed classification into 3 categories: pathogenic (PAT), likely pathogenic (LPAT) and variants of uncertain significance (VUS). None of the variants was assigned as likely benign or benign. CADD v1.3 [40, 41] and REVEL [42] scoring systems were used to predict variant effect (details in the supplement).

### Statistical analysis

Cox’s regression model was used to associate groups with cumulative incidence of disease. Age distribution of groups with different types of *GATA2* variants, cytogenetics, somatic variants and disease group was compared using generalized linear regression model with gaussian link function. Survival rates were estimated using the Kaplan-Meier method. For survival and incidence, endpoints were diagnosis of MDS, GATA2 deficiency or death. In asymptomatic carriers, data were censored at the time of last follow up. Logistic regression model was used to compare differences between groups if the event was discrete. All conducted statistical analyses above were corrected to account for familial relationships within the study cohort by considering the family variable as a clustering variable in the model. Chi-squared test was used to assess if the age distribution of MDS across three age groups are uniformly distributed. *P*-values < 0.05 were considered statistically significant. Data were locked on December 31st, 2023. R Version 4.3.3 (R Foundation for Statistical Computing, Austria) was used for analyses. Data visualization was performed using GraphPad Prism Version 10.2.0 (GraphPad Software, US) and R. Additional details provided in the supplement and figure legends.

## Results

### Hematological phenotypes in the study cohort

In this cohort of 218 patients (Fig. 1A), 205 had symptoms of GATA2 deficiency, while 13 were carriers identified via family analysis and were asymptomatic during last follow-up. Among symptomatic patients, 91.2% (*N* = 187) had MDS and 8.8% (*N* = 18) had cytopenia/immunodeficiency without evidence of MDS (“other cytopenia” group). Within the MDS cohort (187 cases), 120 patients had advanced MDS (80: MDS-EB; 40: RCC/RCMD with -7 cytogenetics) and 67 had early-stage MDS (RCC/RCMD without -7). Advanced MDS diagnosis was overrepresented in pediatric patients compared to adults, consistent with our study’s primary focus on pediatric MDS presentation (*p* = 0.0001, Fig. 1B).

Familial inheritance analysis including both parents was possible in 54 pedigrees. We confirmed de novo status in 35.2% (19/54) and familial inheritance in 64.8% (35/54) of the pedigrees. Familial cases comprised 55 patients and 13 asymptomatic cases across 35 pedigrees. In one family, segregation of the variant, present in 2 siblings and absent in both parents, implied parental mosaicism of germinal or hematopoietic system, phenomena recently described by others [43, 44] (Table S1).

### GATA2-MDS primarily emerges during late childhood and adolescence and is absent in infants

Median age at the time of diagnosis of MDS was 12.9 (range: 1.7-75) years. We observed earlier disease manifestation in patients with advanced MDS versus early-stage MDS across all ages (median 12.4 vs 15.3 years; *p* = 0.009, Fig. 1C, Table 1).

**Table 1:** Clinical and genetic characteristics of the study cohort.

	Age (years)			No. of cases
	Median	IQR	Range	
Total cohort	13.5	10–17.3	1.5–75	218
Hematological phenotype (218 available)				
MDS	12.9	10–16.3	1.7–75	187
Advanced MDS	12.4	9.1–15.2	2.8–75	120
Early-stage MDS	15.3	10.2–18.3	1.7–59	67
Other cytopenia	16	9.7–36.3	1.5-48	18
Asymptomatic carrier	48	19.5–53.5	3–59	13
Immunological phenotypes (192 available)				
Immunodeficiency	13.6	9–17.5	2–59	123
Infections	13.2	8.9–17.5	2–59	105
Mycobacterial infections	24	19–46.2	8.7–59	11
Syndromic features (192 available)				
Lymphedema	12.2	10.9–18	2.8–46.2	27
Deafness	12.6	10.6–16	3.9–46.2	20
Hydrocele	14.2	9.3–17.1	3.1–34	10
Germline *GATA2* variant (218 available)				
Null variant^a^	12.2	9.9–16	1.7–51	115
Other variant	15.3	10–18.8	1.5–75	103
Cytogenetics (192 available)				
-7	12.1	8.7–14.8	2.8-42.7	105
+8	17.1	13.7–21.4	5–75	23
del(5q)	18.8	10.0–35.2	10.8–35.2	3
Normal karyotype	13.7	10.1–17.1	1.5–59	61
Somatic mutations (143 available)				
*SETBP1*	12.2	9.9–14.8	4.4–17.4	37
*ASXL1*	13.2	10.5–16.4	4.4–40.3	31
*STAG2*	16.7	13.7–26.5	8.6–46.2	21
RAS pathway^b^	12.2	10–14.6	4.7–17.4	15
*RUNX1*	11.6	7.3–14.9	4.7–17.4	12
*EZH2*	12.7	8.8-17.4	7.2–20	11
None identified	12.4	9–15.9	2–29	55

^a^Null variants represent those predicted to eliminate or severely disrupt protein function/production and include frameshift truncating, nonsense, splice region and silent variants, and whole gene deletions.

^b^Genes included: KRAS, NRAS, PTPN11, CBL.

*-7* monosomy 7 or der(1;7), *+8* isolated trisomy 8, *IQR* interquartile range (Q1-Q3).

Time to disease analysis was performed only within the consecutive pediatric GATA2-MDS cohort (*N* = 167), to avoid ascertainment bias. It revealed a striking age-dependent pattern of GATA2-MDS where, by age 6 years, only 7.2% of the affected cohort developed MDS, increasing to 45.5% by age 12 years before reaching 100% by the age of 19 years (Fig. 1D). We discovered a distinct age-dependent distribution of MDS: preschool children (0–6 years) represented only 7.2% (*N* = 12), while school-age children (7-12 years) accounted for 38.3% (*N* = 64) and adolescents (13–19 years old) for 54.5% of cases (*N* = 91) (*p* < 0.0001, Fig. 1D). Looking at yearly MDS incidence rates, we found that MDS predominantly emerges from school age onward (Fig. 1E).

Since children with germline predisposition require surveillance, precise data on the youngest GATA2-MDS cases are helpful to devise individualized screening strategies. Therefore, we conducted detailed analysis of the youngest cases with GATA2-MDS. Among preschoolers diagnosed with MDS, 7/12 patients had advanced MDS while 5/12 had early-stage MDS (Table S1). In the 7 cases with advanced MDS, the youngest child was 3 years old (P139, 2.8 years) diagnosed with RCC/-7 necessitating HSCT. The other 6 cases also had -7 and presented between ages 3.9 and 5.5 years. Among the 5 early-stage MDS at preschool age, all were RCC diagnosed at 1.7, 3.1, 3.9, 4 and 5 years, with normal karyotype except for the 5-year-old (P206) having trisomy 8. Importantly, no cases were identified in infants, emphasizing that hematologic malignancy in GATA2 deficiency primarily emerges during late childhood and adolescence.

### Distribution and characteristics of germline GATA2 variants: Enrichment of alterations in ZF2

**Fig. 2:**
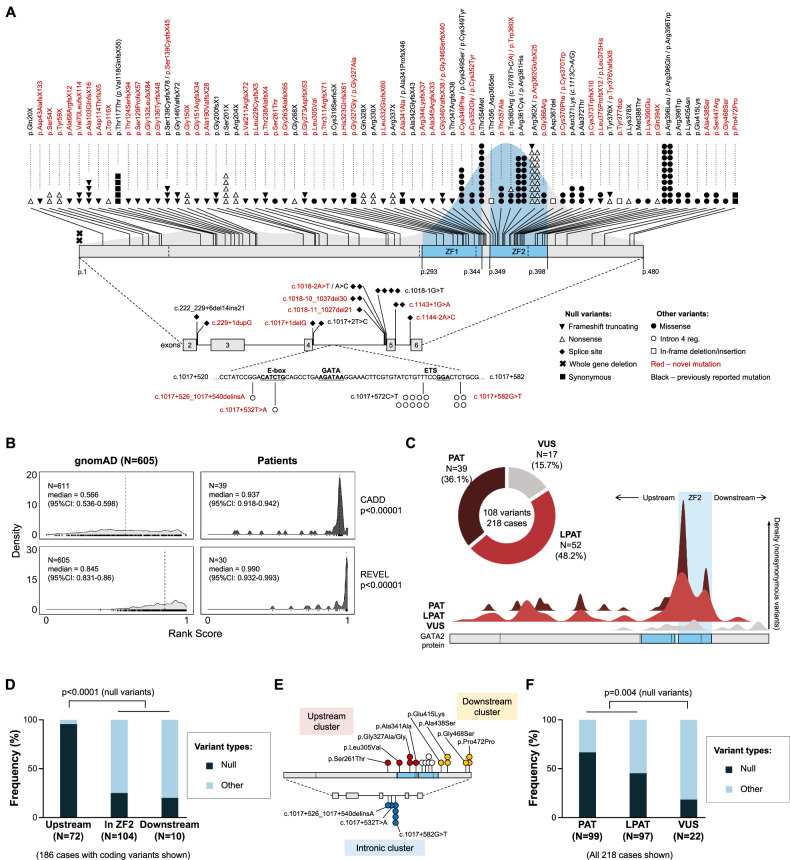
Germline mutational landscape of *GATA2.* **A** Landscape of 108 distinct *GATA2* variants across 218 individuals. 65 variants were not previously reported (red). Most nonsynonymous variants (variants predicted to alter protein structure/function) are localized within the ZF2 domain. Details in Table S2. **B** In silico prediction of *GATA2* variants in our cohort using CADD (*N* = 39) and REVEL (*N* = 30). Distribution of rank scores from computational tools for *GATA2* substitutions shows a distinct clustering of variants in patients compared to control cases from gnomAD. Only substitution variants were included because their effect can be predicted with CADD and REVEL. (ANOVA test used for discrimination between cohorts). Details in Tables S3 and S4. **C** Distribution of variants according to predicted pathogenicity (based on ACMG-AMP evidence codes) and spatial distribution of nonsynonymous variants across GATA2 protein. Details in Tables S2 and S5. **D** Clustering analysis showing differences of variant type localization (*N* = 186 cases with coding variants, Fisher’s exact test). **E** Map of 17 VUSs identified in 22 cases (18 pedigrees). All are novel variants and cluster primarily upstream and downstream of ZF2, and within intron 4. **F** Differences between variant pathogenicity (PAT, LPAT and VUS) and variant types (null and other). The VUS group had significantly more non-null (“other”) variants compared to PAT/LPAT variants (*N* = 218 cases, *p* = 0.004, Fisher’s exact test). LPAT likely pathogenic, PAT pathogenic, VUS variant of unknown significance, ZF2 zinc finger 2.

We identified 3 distinct and previously defined categories of *GATA2* variants: i) null alleles in 52.8% (115/218), comprising 44 frameshift truncating; 44 nonsense; 16 splice region; 9 silent; and 2 whole gene deletions, ii) missense and in-frame indel variants in 40.8% (89/218), and iii) non-coding alterations within intron 4 regulatory region (“intron 4” variants) in 6.4% (14/218) (Fig. 2A). Following the findings by Homan et al. [6], we grouped missense and in-frame variants together under “missense” category (used throughout from now on), due to their similar mutation effect. Among 108 unique germline *GATA2* variants, null alleles were most common (63.9%, 69/108), followed by missense (32.4%, 35/108) and intron 4 variants (3.7%, 4/108). Of the 67 novel (not previously reported) *GATA2* variants, two-third [45] were null alleles, 19 missense and 3 intron 4 variants (Table S2).

The most recurrent variant in our cohort was the nonsense truncating p.Arg362X identified in 18 patients within 16 pedigrees. The p.Arg396 residue emerged as the most frequently affected codon, with p.Arg396Trp/Gln/Leu variants found in 20 cases within 17 pedigrees. The mutational spectrum aligned with the typical human mutational pattern [45], showing a transition-to-transversion ratio of 2.1:1 with C > T substitution being most common (63.1%, 99/157 cases).

Expectedly, most missense variants (77.1%, 27/35) clustered to the ZF2 domain, while 3 (p.Ser261Thr, p.Leu305Val, p.Gly327Ala) were located upstream and 5 downstream (p.Lys405Asn, p.Glu415Lys, p.Ala438Ser, p.Ser447Arg, p.Gly468Ser) of ZF2 (Fig. 2A) [23].

### *GATA2* patient variants are absent or ultrarare in general population and show distinct characteristics

Population frequency analysis demonstrated that most variants (87%, 94/108) were absent from gnomAD database [46]. Among 14 *GATA2* variants found in gnomAD, silent p.Ala341Ala and p.Pro472Pro were rare, with minor allele frequency (MAF) of 0.006% and 0.02%, respectively, while 12 other variants were ultrarare (MAF < 0.005%, Table S2). The relatively high carrier frequency of the 2 silent variants (1:8,767 for p.Ala341Ala and 1:3,420 for p.Pro472Pro), considering the expected high penetrance of GATA2 deficiency suggests these variants are possibly not disease-causing.

We used computational tools to predict deleteriousness of *GATA2* substitution variants in our patients compared to gnomAD controls. Using CADD and REVEL, we found a significant clustering of patient variants towards deleterious scores (Fig. 2B, Tables S3 and S4). Both tools discriminated missense variants found in our patients from those in gnomAD (Fig. 2B). Following the ACMG-AMP criteria, we classified 36.1% (39/108), 48.2% (52/108) and 15.7% (17/108) of the variants as PAT, LPAT and VUS, respectively (Fig. 2C and Tables S2 and S5). We next mapped exonic variants across the protein structure, showing that PAT/LPAT variants clustered together and had highest density within ZF2, while most VUSs were located outside ZF2 (Fig. 2C). Variant localization analysis revealed that null alleles were significantly enriched in the upstream region compared to other variants (p < 0.0001, Fig. 2D), aligning with the PAT/LPAT density peaks upstream of ZF2.

Given the extensive research on GATA2 deficiency in the past decade [6], only few variants remain classified as VUSs. Here, we identified 17 novel VUSs (11 missense, 3 silent and 3 intron 4 variants, Fig. 2E). Compared to PAT/LPAT variants, the VUS group was enriched for non-null (“other”) alleles, which were mostly missense and localized outside of ZF2 (p = 0.004, Fig. 2F). Notably, VUSs observed in 22 individuals across 18 pedigrees were not enriched in asymptomatic carriers compared to symptomatic cases.

### Null variants associate with an earlier onset of MDS and a higher rate of lymphedema and deafness

**Fig. 3:**
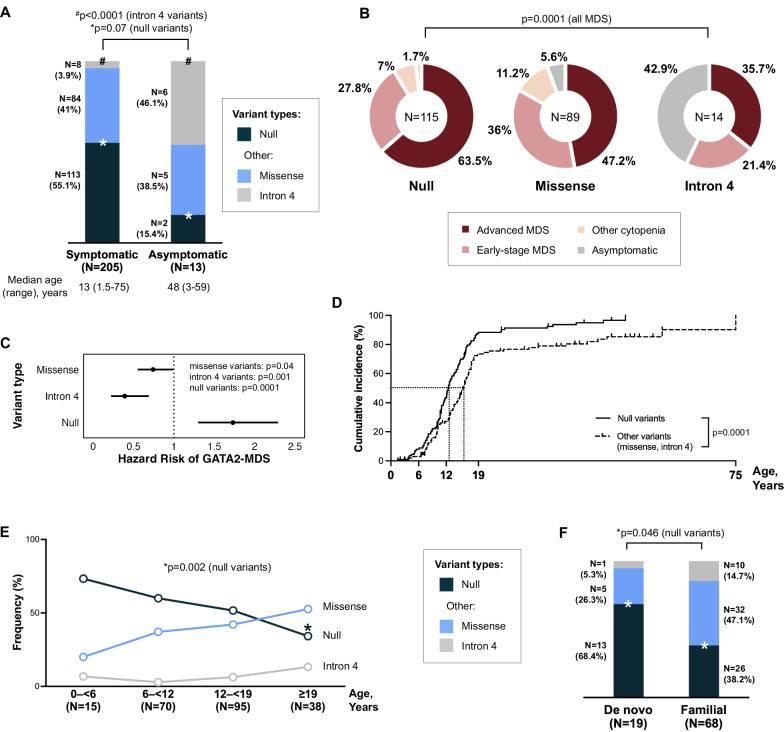
Association of *GATA2* genotypes with clinical phenotypes. **A** Relationship between *GATA2* variant types and disease status. Intron 4 variants were predominantly found in asymptomatic carriers, demonstrating reduced penetrance, while null variants trended toward enrichment in symptomatic individuals (Fisher’s exact test). In-frame variants (2 deletions and 1 insertion) and missense variants (*N* = 86) were analyzed as a combined group. **B** Distribution of hematologic manifestations across *GATA2* variant types. Cox’s regression model was used to compare the proportion of MDS cases (advanced MDS and early-stage MDS) across variant types. **C** Comparative risk assessment for MDS development by variant type (Hazard risk calculation). **D** Cumulative incidence of MDS according to variant type in the total cohort (*N* = 218) (Cox’s regression model). Cases with no MDS diagnosis (other cytopenia and asymptomatic group) were censored at diagnosis time point (corresponding to last follow up). **E** Age-specific prevalence of *GATA2* variants (Fisher’s exact test). **F** Distribution of variant types in familial versus de novo disease (Fisher’s exact test). MDS myelodysplastic syndromes.

Comparing symptomatic patients to asymptomatic carriers, we found that there was a trend for more null variants in symptomatic cases (*p* = 0.07), while missense variants were equally distributed, and intron 4 variants predominated in the asymptomatic group (*p* < 0.0001, Fig. 3A). Next, we analyzed the MDS prevalence by variant type. Patients with null mutations had a higher rate of MDS (*p* = 0.0001, Fig. 3B) which indicated a 1.7-fold increased hazard risk for MDS development (95%CI: 1.3-2.3, Fig. 3C) compared to those with other variants. Similarly, within the null variant group only 1.7% (2/115) were asymptomatic. We confirmed the previous association between null variants and earlier disease onset [8]. In our cohort, null variants correlated with earlier MDS onset compared to other variants (12.2 vs. 14.6 years, respectively, *p* = 0.0001, Fig. 3D) and showed decreasing frequency with age (*p* = 0.002, Fig. 3E). Null variants were enriched in de novo cases compared to familial disease (*p* = 0.046, Fig. 3F), further supporting their high penetrance. We also interrogated non-hematologic phenotypes, available in 192/218 cases (detailed description of phenotypes in supplement). Immunodeficiencies (including infections, autoimmunity and immune cell cytopenia) were reported in 64.1% (123/192), urogenital tract abnormalities in 15.1% (29/192), and respiratory tract abnormalities in 10.9% (21/192). None of these systemic features showed clustering to specific variant types. Lymphedema (*N* = 27) and sensorineural deafness (*N* = 20) were associated with null variants (*p* = 0.01 and *p* = 0.002, respectively, Table S1). While the association between null variants and lymphedema is known [6, 21], the correlation with deafness reported here is new. We did not find any correlations between recurrent missense ZF2 variants and specific phenotypes.

### Intron 4 variants show reduced disease penetrance

Analysis of subjects carrying intron 4 variants showed an association with reduced disease penetrance. Precisely, intron 4 variant carriers were significantly enriched within the asymptomatic group (Fig. 3A) and also had a 0.4-fold decreased hazard risk for MDS (95%CI: 0.2–0.8, *p* = 0.004, Fig. 3C). This attenuated disease phenotype was further supported by the lack of classical lymphedema and deafness in this subgroup, with only 1 patient having hydrocele, hypospadias, and kidney asymmetry as relevant syndromic features. Additionally, 42.9% (6/14) of intron 4 variant carriers remained asymptomatic at last follow-up. These findings suggest an association between intron 4 variants and milder, albeit not absent (since some developed MDS), clinical phenotypes.

### Associated somatic alterations: differences between children and adults

**Fig. 4:**
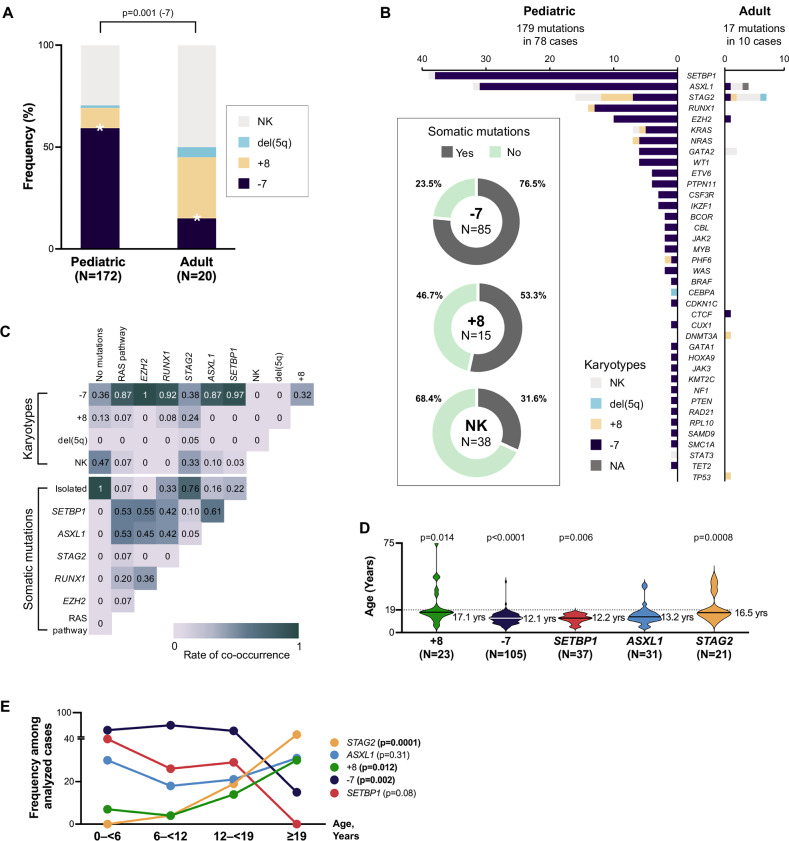
Somatic patterns in GATA2 deficiency. **A** Frequency of karyotype abnormalities across pediatric and adult patients. **B** Somatic mutation burden and its relationship with karyotypes. Horizontal axis represents the number of mutations identified. Within the box, proportion of patients carrying somatic mutations across 3 predominant karyotype groups are shown, as indicated in the legend. **C** Co-occurrence matrix of somatic mutations and karyotypes. For each somatic event (columns) rates of co-occurrence with other cytogenetic changes and somatic mutations (rows) are shown. **D** Age at diagnosis in patients with common somatic alterations (Non-parametric Mann-Whitney U test used to compare median age at diagnosis for each somatic alteration, bolded p values are significant). **E** Prevalence of somatic alterations across age groups (Linear regression model, bolded p values are significant). Youngest patients with abnormal karyotype (monosomy 7): 2.8 and 3.9 years old (RCC). Youngest patient with somatic mutations: 4.4 years old (MDS-EB). Details in Table S6. -7 monosomy 7, +8 trisomy 8, NA not available, NK normal karyotype.

In our cohort, -7 was the most prevalent cytogenetic lesion observed in 54.7% (105/192) of patients, followed by isolated trisomy 8 ( + 8) in 12% (23/192) and normal karyotype in 31.8% (61/192) of cases (Table S1). Expectedly, -7 was significantly enriched in pediatric compared to adult cohort (*p* = 0.001) (Fig. 4A, Table 1). While knowledge on clonal hematopoiesis in GATA2 deficiency is evolving, data on age-related differences is lacking. Hence, we performed comprehensive analysis of somatic mutational landscape. We identified somatic mutations in 60% (78/130) of the symptomatic pediatric and 76.9% (10/13) adult cases undergoing clinical somatic testing. We found 196 mutations across 38 genes (Fig. 4B, Table S6). Recurrently mutated genes were *SETBP1* (25.9%, 37/143 cases), *ASXL1* (21.7%, 31/143), *STAG2* (14.7%, 21/143), *RUNX1* (8.4%, 12/143), *EZH2* (7.7%, 11/143), *KRAS* (4.9%, 7/143), *GATA2* (4.2%, 6/143), *NRAS* (4.2%, 6/143), *WT1* (3.5%, 5/143), *PTPN11* (2.8%, 4/143), *ETV6* (2.1%, 3/143), *CSF3R* (2.1%, 3/143), *IKZF1* (2.1%, 3/143), *BCOR* (1.4%, 2/143), *CBL* (1.4%, 2/143), *JAK2* (1.4%, 2/143), *MYB* (1.4%, 2/143), *PHF6* (1.4%, 2/143) and *WAS* (1.4%, 2/143). Single mutations were found in 19 additional genes spanning diverse cellular pathways including *BRAF*, *CEBPA, CDKN1C, CTCF, CUX1, DNMT3A*, *GATA1*, *HOXA9*, *JAK3*, *KMT2C*, *NF1*, *PTEN*, *RAD21*, *RPL10*, *SAMD9*, *SMCA1*, *STAT3, TET2* and *TP53*.

Analysis of somatic mutational patterns pointed to a unique association with -7: all recurrent mutations, (except for *STAG2*) co-occurred with -7, which was present in 73.9% (65/88) of mutation-positive cohort. Furthermore, -7 cases exhibited the highest mutation frequency (76.5%, 65/85) compared to isolated +8 (53.3%) and normal karyotype group (31.6%) (Fig. 4B). Virtually all patients carrying *SETBP1* mutation (36/37) had -7 clone, suggesting a synergistic relationship in malignant progression. Similarly, all 9 cases with *EZH2* mutations and most (27/31) *ASXL1*-mutated patients harbored -7. Mutation co-occurrence analysis revealed that *SETBP1, ASXL1, EZH2, RUNX1* and members of the RAS pathway (*KRAS, NRAS, PTPN11, CBL*) frequently occurred together (Fig. 4C).

Recently, *STAG2* mutations were implicated as frequent somatic changes in clinically stable GATA2 patients suggesting that they may improve hematopoietic cell fitness [8]. In our cohort we detected 23 *STAG2* mutations in 21 patients who were significantly older (median 16.7 years) compared to cases with other somatic alterations (*p* = 0.0008, Fig. 4D). In contrast, *SETBP1* mutations and -7 alterations were associated with younger age.

**Fig. 5:**
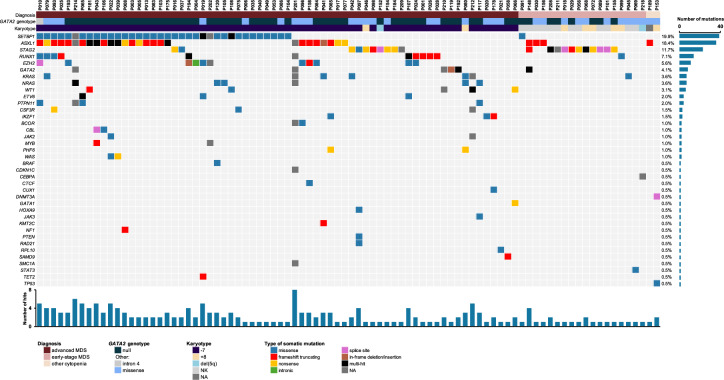
Somatic landscape of patients with GATA2 deficiency. Molecular characterization identified somatic mutations in 88/143 patients. Complete dataset available in Table S6. -7 monosomy 7, +8 trisomy 8, NA not available, NK normal karyotype.

To gain insights into temporal patterns of clonal progression, we plotted the prevalence of somatic alterations across age. Distribution of *SETBP1* mutations followed -7, with highest frequency in younger age groups (Fig. 4E). In contrast, *STAG2* mutations showed a steady increase in frequency, similar to +8, while *ASXL1* mutations were comparable across age groups (Fig. 4E, Table 1). The complete landscape of somatic mutations is shown in Fig. 5.

## Discussion

This comprehensive analysis of 218 individuals with germline *GATA2* variants, focused on the natural history of pediatric MDS, reveals unique age-dependent patterns of disease evolution and establishes novel genotype-phenotype correlations with implications for clinical surveillance. Most notably, we demonstrate that advanced MDS is virtually absent before age 3 years. Furthermore, the remarkably low incidence of MDS (including advanced MDS and early-stage MDS) in early childhood (7.2% of the cohort by age 6 years) with a steep increase during school-age, reaching 45.5% by age 12 years suggests that early hematopoiesis remains relatively preserved despite GATA2 deficiency. This observation is consistent with findings from several *Gata2* haploinsufficient mouse models showing delayed phenotypic manifestation with cytopenia emerging in older mice and under proliferative stress [47–49]. From a clinical management perspective, these results change our understanding of disease evolution and provide an evidence base for refined surveillance protocols. The negligible risk of MDS in very young children suggests that invasive bone marrow examinations are not justified in otherwise healthy pre-school children which can be safely monitored with complete blood counts (CBC). The increasing incidence of MDS during school age supports the initiation of systematic hematologic monitoring during this period. However, the optimal trigger for initiating BM exams requires careful evaluation – on one hand, cytopenia might serve as a trigger for BM evaluation, on the other hand some patients could develop MDS without relevant peripheral blood count abnormalities thus justifying regular marrow surveillance from school age onward regardless of CBC findings. As more asymptomatic carriers with *GATA2* mutations are being identified through family screening, prospective evaluation of these individuals at risk will be essential to determine the most appropriate surveillance strategy.

Through detailed variant analysis, we identified 108 unique germline *GATA2* variants, with majority classified as PAT or LPAT and only 15.7% classified as VUS. We found a distinctive clustering, with VUSs (most being missense variants) localizing outside the ZF2 domain, while PAT/LPAT variants expectedly showed significant enrichment within ZF2. Although the true clinical relevance of the 17 VUSs affecting 22 individuals remains unclear at this time, associated phenotypes were consistent with GATA2 deficiency. We note that variant classification based on the generic ACMG criteria might not fully support the nuanced analysis of novel *GATA2* variants, pointing out the need for gene-specific modifications, similar to those implemented for *RUNX1* [50, 51].

Several key genotype-phenotype associations emerged from our correlative analyses. In this pediatric-skewed cohort, null variants (found in half of the cases) confer a 1.7-fold increased hazard risk for MDS development compared to other variant types and are enriched in symptomatic cases, particularly those with MDS. The association between null variants and earlier disease onset provides a rationale for more vigilant surveillance in this genotype subgroup. Future studies should evaluate whether patients with null alleles might benefit from early systematic surveillance and potentially even preemptive HSCT. Additionally, we identified a previously unreported association between null variants and deafness, expanding the spectrum of genotype-phenotype correlations. A particularly striking finding is the first clear evidence for reduced disease penetrance in patients with intron 4 regulatory variants. We found that over 40% of these individuals remained asymptomatic and showed no manifestations of lymphedema and deafness. This finding may justify adapted strategy for carrier counseling for this specific genetic subgroup, as a substantial proportion of these patients in our cohort remained without MDS until older age.

Our analysis of the somatic mutational landscape, based on the largest cohort of GATA2-MDS to date, revealed distinct GATA2-specific and age-dependent patterns of clonal hematopoiesis. The most recurrently mutated genes were *SETBP1*, *ASXL1*, *STAG2*, *RUNX1*, *EZH2* and the RAS pathway genes. We found that -7 emerged as a “central hub” associating with somatic mutations, with 76.5% of -7 cases harboring additional somatic mutations. Conversely, we found that most (73.9%) of the patients with somatic mutations have co-occurring -7. There was a near-perfect correlation between *SETBP1* mutations and -7 (36/37 cases) suggesting that *SETBP1* is a molecular surrogate for -7 clone that defines high-risk disease in GATA2 deficiency, with immediate implications for timely HSCT and molecular monitoring post HSCT. We observed age-dependent dynamics of clonal hematopoiesis: both *SETBP1* mutations and -7 showed decreasing frequency with advancing age. *STAG2* mutations and trisomy 8 demonstrated an opposing pattern with increasing prevalence in older patients. These clonal events likely represent pre-malignant changes with indeterminate potential that may initially provide a compensatory mechanism. Patients with *STAG2* mutations were older and had less co-occurring somatic events compared to the other somatic subgroups. This aligns with the findings by Largeaud and colleagues who found that *STAG2* mutated patients with GATA2 deficiency were older and had almost no other somatic events; they also found enrichment of multiple *STAG2* mutations per patient [8]. The authors proposed that *STAG2* mutations may confer selective advantage to GATA2-deficient hematopoietic cells and function more as a rescue rather than transformative events.

Our findings must be interpreted in light of several limitations. First, our cohort was enriched for pediatric cases with a younger age at presentation (median 13.5 years), compared to previous studies reporting median ages from 16 to 26 years [7, 8, 20]. However, the focus on pediatric GATA2-MDS also offered an opportunity to study phenotypes in a homogeneous MDS cohort where variant effects are more pronounced. Second, variable follow-up duration, particularly for asymptomatic carriers, means that lifetime penetrance estimates require validation through longitudinal studies. The focus on hematologic phenotypes may have led to underreporting of some syndromic phenotypes, particularly in adult cases. Cases with early-stage MDS and cytopenia without clear MDS diagnosis demonstrate phenotypic similarities, however, the inherent limitations of our study—including variations in diagnostic criteria across submitting countries and potentially insufficient granular data—prevent confidently grouping these two diagnostic categories together. Finally, while our results provide new insights into age-dependent patterns of clonal hematopoiesis in GATA2-MDS, our targeted somatic testing did not include all possible genes associated with clonal hematopoiesis and additional studies with larger adult cohorts would be valuable to extend these findings across full age spectrum.

Looking ahead, our findings raise important questions about the mechanisms underlying the age-dependent emergence of GATA2-MDS. The sharp increase in incidence during school age suggests this period represents a critical window for malignant transformation. Clonal hematopoiesis in GATA2 deficiency shows distinct age-dependent patterns - evidenced by the varying frequencies of both low-risk lesions (like *STAG2*) and high-risk events (like -7 or *SETBP1*) across different age groups. Understanding the biological basis for these unique patterns could open avenues for personalized therapy and monitoring strategies.

In conclusion, this study provides a framework for genotype-based risk stratification in GATA2 deficiency and suggests age-appropriate surveillance strategies. Future prospective studies will be valuable in validating our findings and further improving genotype-driven recommendations, especially regarding the optimal timing of HSCT and the most effective long-term surveillance strategies.

## Supplementary information

Supplement Material

Readme for Supplement Tables

Supplement Table 1

Supplement Table 2

Supplement Table 3

Supplement Table 4

Supplement Table 5

Supplement Table 6

Supplement Table 7

## Data Availability

Data on germline and somatic genetic variants and phenotype characterization are deposited in the Supplemental Tables. Sequencing data files are available upon request from corresponding author.
