# Supplementary material for: Age-dependent phenotypic and molecular evolution of pediatric MDS arising from GATA2 deficiency
Source: Blood Cancer J. 2025 Jul 15;15(1):121. doi: 10.1038/s41408-025-01309-6 (PMC12264048; doi:10.1038/s41408-025-01309-6)
Supplement: Supplementary file 1 — Supplement Material [file 41408_2025_1309_MOESM1_ESM.docx]

**Age-dependent phenotypic and molecular evolution of PEDIATRIC MDS ARISING FROM GATA2 DEFICIENCY**

**Supplemental Methods**

*Somatic studies*

Targeted deep sequencing (Ampliseq panel #IAD51150_150, ThermoFisher Scientific, TN, US, NEBNext Direct panel #N7060B-X1AJJ, New England Biolabs, MA, US, AmpliSeq panel v2 #IAA11909_192, ThermoFisher Scientific) libraries were prepared following PCR enrichment using the NEBNext® Ultra^TM^ II DNA Library Prep Kit for Illumina (New England Biolabs) according to the manufacturer’s instructions and were sequenced on a MiSeq Sequencer platform (Illumina, CA, US) using 150 bp paired-end chemistry. The text-based output FASTQ files were mapped and aligned to hg19 reference genome using the SeqPilot/SeqNext v.4.40 software (JSI Medical Systems, Germany) followed by quality assessment and subsequent variant annotation. FASTQ files were aligned to the hg19 genome with Burrows-Wheeler Aligner 20 with further data processing and annotation performed with ANNOVAR according to Genome Analysis Toolkit guideline as previously published^1-4^.

Initial variant calling during targeted deep sequencing was restricted to quality score of 30 (Q30, 99.9% base call accuracy) and a minimum absolute coverage of 30 reads to abate incorrect base calls. A minimum coverage of 150x was applied for variants with a minimum variant allele frequency (VAF) of 5%. Other quality thresholds were employed as recommended by the Illumina Technical Notes (Quality Scores for Next-Generation Sequencing, 2011, Somatic Variant Caller, 2012). Variants were visualized by SeqPilot/SeqNext v.4.40 and Alamut Visual (v.2.8-2.10, Interactive Biosoftware, Rouen, France).

*Variant pathogenicity assessment (ACMG-AMP variant classification for germline GATA2 variants)*

Pathogenicity assessment of the germline *GATA2* variants was performed according to the guidelines of the American College of Medical Genetics and Genomics and Association for Molecular Pathology (ACMG-AMP). Variants were assigned to pathogenic, likely pathogenic and variant of unknown significance categories based on the ACMG-AMP criteria^5^. Missense variant effect was determined using prediction scores from REVEL^6^. For variants predicted to affect splicing, SpliceAI^7^ was applied to assess splice gains/losses. Locus conservation across species was assessed using PhyloP^8,9^ (**Table S2**).

Specifically, the following ACMG-AMP evidence codes were applied to the uncovered *GATA2* variants:

- PVS1 for all null variants (frameshift truncating, nonsense/stop gain, synonymous RNA deleterious variants, splice region, whole exon/gene deletions)
- PS1 for missense variants with same amino acid change reported as pathogenic in other studies.
- PS2_Moderate for confirmed de novo variants.
- PS3 for variants with functional studies supportive of loss-of-function effect.
- PS4 for variants significantly enriched in ≥2 affected individuals compared to control population. We applied PS4_Moderate for variants with single cases in our cohort. Significance was calculated using Fisher’s exact test with Bonferroni correction to compare the prevalence of variants in our cohort to ~800,000 cases from the gnomAD v4.1.0 population database (**Table S5**).
- PM1 for variants located in the zinc finger 2 domain.
- PM2_Supporting for variants absent from controls in the gnomAD database or classified as ultra-rare (i.e. minor allele frequency lower than 0.005%).
- PM4 for all in-frame variants (in frame deletions / insertions).
- PM5 for missense variants at an amino acid residue where a different change has been previously established as pathogenic.
- PP1 for variants in cases with confirmed familial origin of disease.
- PP3 for variants with computational evidence supporting pathogenicity. PP3 was assigned to all missense variants with REVEL score >0.640, except for nonsense variants, where PP3 was not applied. PP3 was assigned to all splice variants with SpliceAI Δ score >0.5. PP3 was not applied for variants that meet criteria for PVS1.
- BP4 for variants where computational predictions indicate that the variant is unlikely to affect gene function. BP4 was assigned to missense variant with REVEL score <0.290 or intronic variant outside of the natural splicing region (+/- 1 or 2) with SpliceAI Δ score <0.2.
- BP7 for silent variants with SpliceAI Δ score <0.2 and low nucleotide conservation indicated by phyloP. It can also be applied to intronic variants at or within ±7 to ±21 locations.

The following ACMG-AMP criteria were not utilized during variant classification:

- PM6 for assumed de novo variants since due to incomplete penetrance it is not possible to ascertain based on phenotype alone if variant is de novo.
- PP2 for missense variants because of the insignificant constraint of GATA2 for missense variation based on gnomAD population data where observed is close to expected frequency (Z-score 1.73).
- PP5 for variants reported as pathogenic by reputable sources without conflicting evidence, based on international consensus.
- BS2 for variants observed in asymptomatic kindreds due to the possibility of delayed disease onset in asymptomatic mutation carriers.
- BP6 for variants reported as benign by reputable sources without conflicting evidence, based on international consensus.

*Statistical analysis*

(Specifications in response to the reporting summary).

Due to the retrospective nature of our study, the sample size was limited to the patient population who met our inclusion criteria (outlined in the Material and Methods section). We did not calculate the statistical power. Our analysis of the somatic mutational landscape is based on the largest cohort of GATA2-MDS to date. The statistical tests are justified as appropriate for all figures and tables presented in this study. Prior to conducting any statistical analyses, we verified that the data met the assumptions required for each test. For continuous variables, data normality was assessed using the Shapiro-Wilk test to determine appropriate statistical approaches. We estimated the variance of the data within each group to account for data distribution. When comparing between groups, we checked if the variance is similar between the groups that are being statistically compared. If not, we used the robust standard error based on the linear regression model to ensure statistical validity.

**Supplemental Results**

*Clinical phenotypes in the study cohort*

All symptomatic patients (N=205) presented with abnormally low blood counts on CBC and/or immune cell cytopenia. Recurrent/persisting infections were reported in 54.7% (105 of 192 cases with evaluable data): viral in 76, non-mycobacterial in 52, mycobacterial in 11 and fungal in 10 cases. Lymphedema and sensorineural deafness were observed in 14.1% (27/192) and 10.4% (20/192) evaluable cases, respectively. Respiratory tract involvement was observed in in 10.9% (21/192) and included pulmonary alveolar proteinosis (confirmed in 2 patients and suspected in another) and other respiratory tract abnormalities including bronchial asthma, acute respiratory distress syndrome and interstitial lung disease. Urogenital tract disorders affected 15.1% (29/192) of the cohort. Patient IDs are listed in **Table S7**.

**References**

1. McKenna A, Hanna M, Banks E, et al. The Genome Analysis Toolkit: a MapReduce framework for analyzing next-generation DNA sequencing data. *Genome Res*. 2010;20(9):1297-1303.

2. Wang K, Li M, Hakonarson H. ANNOVAR: functional annotation of genetic variants from high-throughput sequencing data. *Nucleic Acids Res*. 2010;38(16):e164.

3. Van der Auwera GA, Carneiro MO, Hartl C, et al. From FastQ data to high confidence variant calls: the Genome Analysis Toolkit best practices pipeline. *Curr Protoc Bioinformatics*. 2013;43(1110):11 10 11-11 10 33.

4. Pastor VB, Sahoo SS, Boklan J, et al. Constitutional SAMD9L mutations cause familial myelodysplastic syndrome and transient monosomy 7. *Haematologica*. 2018;103(3):427-437.

5. Richards S, Aziz N, Bale S, et al. Standards and guidelines for the interpretation of sequence variants: a joint consensus recommendation of the American College of Medical Genetics and Genomics and the Association for Molecular Pathology. *Genet Med*. 2015;17(5):405-424.

6. Ioannidis NM, Rothstein JH, Pejaver V, et al. REVEL: An Ensemble Method for Predicting the Pathogenicity of Rare Missense Variants. *Am J Hum Genet*. 2016;99(4):877-885.

7. Jaganathan K, Kyriazopoulou Panagiotopoulou S, McRae JF, et al. Predicting Splicing from Primary Sequence with Deep Learning. *Cell*. 2019;176(3):535-548 e524.

8. Pollard KS, Hubisz MJ, Rosenbloom KR, Siepel A. Detection of nonneutral substitution rates on mammalian phylogenies. *Genome Res*. 2010;20(1):110-121.

9. Kent WJ, Sugnet CW, Furey TS, et al. The human genome browser at UCSC. *Genome Res*. 2002;12(6):996-1006.
